# Shikonin Suppresses Skin Carcinogenesis via Inhibiting Cell Proliferation

**DOI:** 10.1371/journal.pone.0126459

**Published:** 2015-05-11

**Authors:** Wenjuan Li, Chunjing Zhang, Amy Ren, Teena Li, Rong Jin, Guohong Li, Xin Gu, Runhua Shi, Yunfeng Zhao

**Affiliations:** 1 Department of Pharmacology, Toxicology & Neuroscience, LSU Health Sciences Center in Shreveport, Shreveport, Louisiana, United States of America; 2 Department of Neurosurgery, LSU Health Sciences Center in Shreveport, Shreveport, Louisiana, United States of America; 3 Department of Pathology, LSU Health Sciences Center in Shreveport, Shreveport, Louisiana, United States of America; 4 Feist-Weiller Cancer Center, LSU Health Sciences Center in Shreveport, Shreveport, Louisiana, United States of America; Southern Illinois University School of Medicine, UNITED STATES

## Abstract

The M2 isoform of pyruvate kinase M2 (PKM2) has been shown to be up-regulated in human skin cancers. To test whether PKM2 may be a target for chemoprevention, shikonin, a natural product from the root of *Lithospermum erythrorhizon* and a specific inhibitor of PKM2, was used in a chemically-induced mouse skin carcinogenesis study. The results revealed that shikonin treatment suppressed skin tumor formation. Morphological examinations and immunohistochemical staining of the skin epidermal tissues suggested that shikonin inhibited cell proliferation without inducing apoptosis. Although shikonin alone suppressed PKM2 activity, it did not suppress tumor promoter-induced PKM2 activation in the skin epidermal tissues at the end of the skin carcinogenesis study. To reveal the potential chemopreventive mechanism of shikonin, an antibody microarray analysis was performed, and the results showed that the transcription factor ATF2 and its downstream target Cdk4 were up-regulated by chemical carcinogen treatment; whereas these up-regulations were suppressed by shikonin. In a promotable skin cell model, the nuclear levels of ATF2 were increased during tumor promotion, whereas this increase was inhibited by shikonin. Furthermore, knockdown of ATF2 decreased the expression levels of Cdk4 and Fra-1 (a key subunit of the activator protein 1. In summary, these results suggest that shikonin, rather than inhibiting PKM2 in vivo, suppresses the ATF2 pathway in skin carcinogenesis.

## Introduction

Shikonin is an active component isolated from *lithospermum erythrorhizon*, a traditional oriental medicinal herb, which has been used to treat HIV-1 infection [1]. Shikonin’s anti-tumor activity has been studied since the 1990s. Shikonin and its derivatives have been shown to induce cell death in a variety of human cancer cells [2–9]. In addition, shikonin and its derivatives also show anti-angiogenesis [10,11], anti-inflammation [12], and anti-glycolysis activities [13,14]. Although the chemopreventive activity of shikonin has been suggested in a rat intestinal carcinogenesis study [15], the potential application of this compound in chemoprevention has not been thoroughly investigated.

Targeting cancer metabolism for cancer therapy and prevention is one emerging research topic. Many types of cancer cells predominantly produce ATP by higher rates of glycolysis followed by lactic acid fermentation even with ample oxygen, known as the “Warburg Effect”. Pyruvate kinase catalyzes the last step of glycolysis and the M2 isoform PKM2, is highly expressed in cancer cells [16]. However, how to target PKM2 in cancer treatment is not conclusive, since both inhibition and activation of PKM2 have been shown to suppress cancer cell growth [17]. This is at least partially due to the fact that in cancer cells, PKM2 can exist as either a tetramer with higher activities or a dimer with lower activities. In addition, PKM2 can translocate to nucleus and exerts both kinase and non-kinase activities [17].

We aim to test if targeting PKM2 can prevent tumor promotion, the early stage of carcinogenesis. Shikonin has been recently identified as a specific inhibitor to PKM2 [13]. Since PKM2 is highly expressed in many types of human carcinoma including skin cancers, shikonin anti-tumor promotion activity may involve regulation of PKM2 activity as it was suggested by a recent study. Using a promotable skin epidermal JB6 cell model, this study showed shikonin suppressing tumor promoter-induced PKM2 activation and glycolysis without inducing apoptosis [14]. To further investigate the potential chemopreventive activity of shikonin, we performed a multistage skin carcinogenesis study.

## Materials and Methods

### Cell lines, Reagents and Treatment

Murine skin epidermal JB6 Cl-41 (P+) cells (purchased from American Type Culture Collection) were used to study tumor promotion. Cells were grown in EMEM medium containing 4% fetal bovine serum (FBS), 2 mM L-glutamine, and 2.5 μg/ml penicillin and 2.5 μg/ml streptomycin in a 37°C incubator under 5% CO_2_.

For cell culture, the levels of mycoplasma were routinely (once every three months) assessed in these cells using a MycoAlert Mycoplasma Detection Kit purchased from Lonza (Rockland, ME), and the results were consistently negative.

Shikonin (SKN), purchased from Sigma (S7576), was dissolved in dimethyl sulfoxide (DMSO, Sigma, St. Louis, MO). The chemical carcinogen dimethylbenz[α]anthracene (DMBA, Sigma) and the tumor promoter 12-O-tetradecanoylphorbol-13-acetate (TPA, Sigma) were also prepared in DMSO.

### Chemically-induced mouse skin carcinogenesis

Sixty 6–8-week old female DBA/2 mice (which are relatively sensitive to skin carcinogenesis) from Jackson Laboratory (Indianapolis, IN) were divided into 4 groups: DMSO, DMBA/TPA, SKN, SKN+DMBA/TPA. The DMSO group (5 mice) received DMSO treatment as the vehicle control; the DMBA/TPA group received a single topical application of 200 nmol DMBA for 2 weeks, following by a single topical application of 5 μg TPA (12-*O*-tetradecanoylphorbol-13-acetate), once per day, three times per week for 14 weeks. The SKN group received topical application of shikonin at 10 μg following the same schedule for DMBA/TPA treatments. The SKN+DMBA/TPA groups received shikonin (SKN) treatment first followed by TPA treatment 30 min later. At the end of the skin carcinogenesis study, mice were euthanized by pentobarbital (150 mg/kg, i.p.). The skin samples from experimental sites were collected and submitted for biochemical and morphologic analysis as described in the following. This animal protocol was approved by the Animal Care and Use Committee of LSU Health Sciences Center in Shreveport.

### Counting apoptotic and mitotic cells

Skin samples were fixed in 4% buffered formaldehyde, embedded in paraffin and processed for hematoxylin eosin (H&E), Ki-67 (Abcam, ab16667) and TUNEL (Roche, #11684817910) staining. Mitoses and apoptotic cells were counted using light microscopic evaluation of H&E stained slides. Morphologic features, such as cell shrinkage, formation of cytoplasmic vacuoles were used to identify apoptosis. Morphologic analysis was performed by one of the authors (XG), a board-certified pathologist. TUNEL and Ki-67 staining was performed following the manufacturer’s instructions. The secondary antibodies were conjugated with Alexa Fluor 488 dye and the images were taken using a Nikon fluorescence microscope.

### Preparation of whole cell lysate

Collected cultured skin cells were suspended in 150 μl of PBS containing a proteinase inhibitor cocktail (Calbiochem, La Jolla, CA). Cells were sonicated on ice for two strokes (10 sec per stroke) using a Fisher Sonic Dismembrator (Model 100, Scale 2). After incubating on ice for 30 min, cell lysate was centrifuged at 14,000 x g for 15 min, and the supernatant was collected and designated as Whole Cell Lysate.

### PKM2 expression and activity assay

PKM activities were analyzed using the lactate dehydrogenase (LDH)-coupled assay as described previously [18,14] and Whole Cell Lysate was used for the assay.

### Antibody microarray analysis

The Panorama Antibody Microarray Cell Signaling kit (Catalog Number CSAA1-1, Sigma-Aldrich) was used. The array kit was composed of 224 highly specific antibodies spotted in duplicate on nitrocellulose-coated glass slides. Each antibody microarray contained 32 subarrays with duplicate spots of seven antibodies as well as a single positive control for Cy3 and Cy5 and a single negative control. The list of arrayed antibodies can be found at the Sigma-Aldrich’s website. Whole Cell Lysate from each treatment group was pooled together and used for the assay. The experiments were performed following the instructions provided by the manufacturer.

### Knockdown of AFT2 by siRNA

JB6 P+ cells were seeded (2 x 10^5^ cells/well) in six-well tissue culture plates, and incubated at 37°C in a 5% CO2 incubator until becoming 70–80% confluent. For each transfection, 2 μl of ATF2 siRNA duplex (sc-29756, Santa Cruz Biotechnology, Santa Cruz, CA) was diluted into 100 μl of siRNA transfection medium (sc-36868, Santa Cruz Biotechnology). In a separate tube, 2 μl of transfection reagent (sc-29528, Santa Cruz Biotechnology) was diluted into 100 μl of siRNA transfection medium. The dilutions were mixed gently together and incubated for 30 min at room temperature. Fluorescein conjugated control siRNA (sc-36869, Santa Cruz Biotechnology) was used to monitor the transfection efficiency, which was approximately 70%.

### Western blot analysis

Whole Cell Lysate was used for the assay. Antibodies against ATF2 (sc-187), Fra-1 (sc-605), α-Tubulin (sc-5286), and β-Actin (sc-47778) were purchased from Santa Cruz Biotechnology.

### Statistical analysis

Data were presented as mean ± standard division (S.D.). The Fisher’s Exact Test and student’s t-test were used to compare the tumor incidence and multiplicity, respectively. Statistical analyses was performed by using SAS 9.3 (SAS, Gary, NC). All p values less than 0.05 were considered as statistically significant.

## Results

### Shikonin suppressed chemically-induced skin carcinogenesis

At the end of the skin carcinogenesis study, animals’ bodyweights were first measured and the data were presented in the following order: DMSO, SKN, DMBA/TPA, and SKN+DMBA/TPA: 22.1±2.0g, 22.1±1.9g, 22.0±1.4g, and 21.9±1.4g. Results from statistical analysis revealed that neither shikonin (p = 0.87) nor chemical carcinogen treatment (p = 0.37) affected the animal’s bodyweight.

Skin papilloma formation was examined by a pathologist, and the results are summarized in Table 1. The tumor incidence of the SKN+TPA group (73.3%) was not significantly decreased compared with that of the carcinogen treatment group (93.3%) (p = 0.329 by two sided Fisher's Exact Test). However, the tumor multiplicity in the SKN+TPA group (1.6±1.7) was significantly decreased (p = 0.0035 by student’s t-test) compared with the carcinogen treatment group (3.9±2.3).

**Table 1:** Papilloma formation in the multistage carcinogenesis model.

Treatment	Number of mice	Tumor Incidence	Papillomasper mouse	Total Papillomas
DMSO	5	0%	0±0	0
TPA	15	93.3%^#^	3.9±2.3	59
SKN	5	0%	0±0	0
SKN+TPA	15	73.3%^#^	1.6±1.7*	24

DMSO, DMSO-treated group; TPA, DMBA/TPA-treated group; SKN, shikonin-treated group; SKN+TPA, shikonin plus DMBA/TPA-treated group.

^#^p<0.01 compared with its own control group by Fisher’s Exact Test;

*p<0.01 compared with the TPA group by student’s t-test.

**Fig 1:**
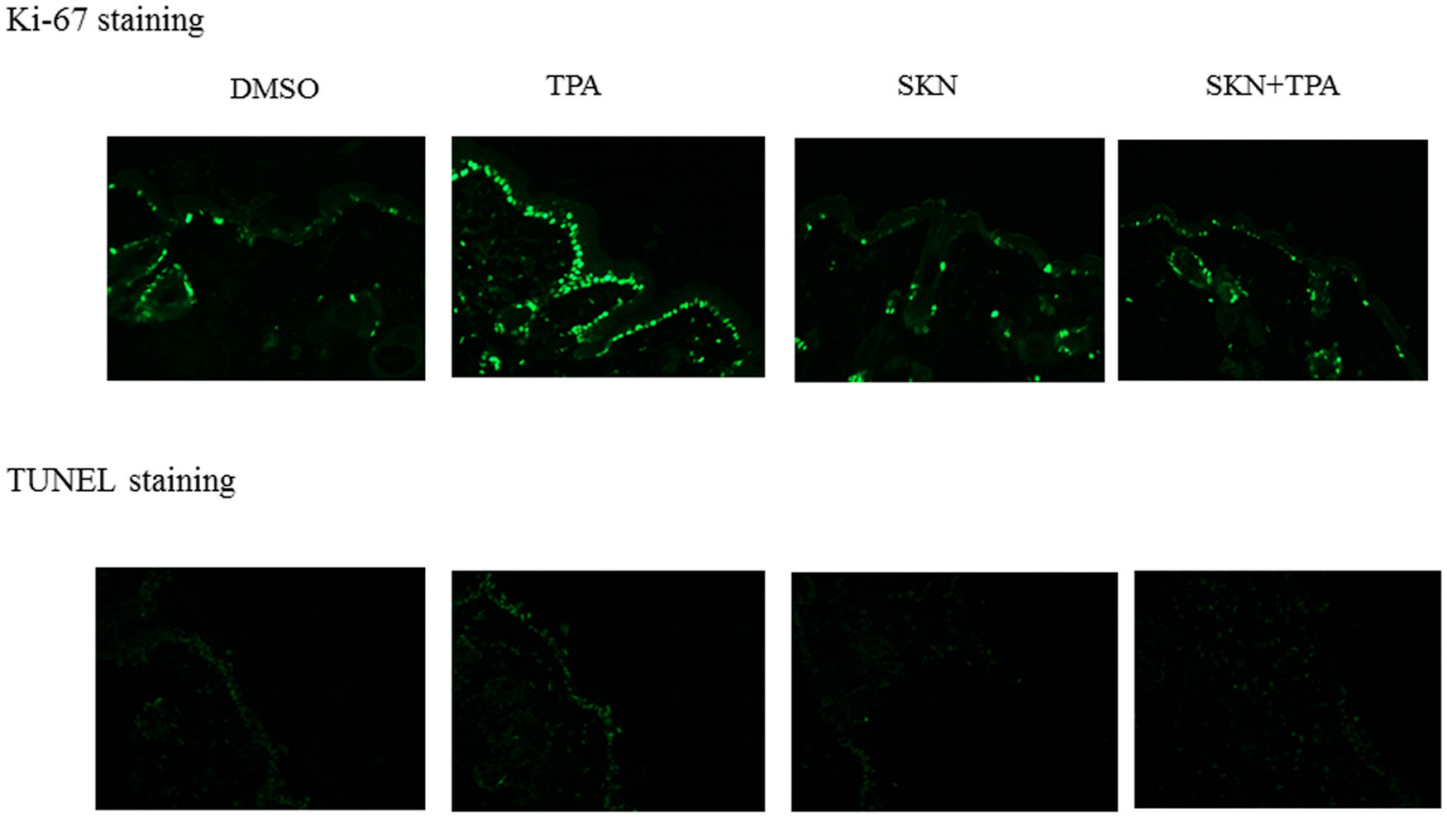
Ki-67 and TUNEL staining of skin epidermal tissues at the end of the skin carcinogenesis study (amplification: 20x). Ki-67 and TUNEL staining was performed according to the manufacturer’s instructions. A representative image from each treatment group was shown. DMSO, DMSO-treated group; TPA, DMBA/TPA-treated group; SKN, shikonin-treated group; SKN+TPA, shikonin plus DMBA/TPA-treated group.

Does shikonin inhibit cell proliferation or cause cell death to exert its tumor preventive effect? Ki-67 and TUNEL staining of the skin epidermal tissues was performed and the results were shown in Fig 1. Higher levels of Ki-67/TUNEL staining were observed in the carcinogen group (TPA), whereas lower levels of Ki-67/TUNEL staining were observed in the three other groups. Mitotic figures and apoptotic cells were then counted precisely using H&E stained tissue sections. As summarized in Table 2, cell mitosis and apoptosis were increased by 1.8 and 1.6 fold, respectively, in the TPA group comparing with the DMSO group, which is similar to what has been observed in our previous studies [19,20].These increases were suppressed by topical application of shikonin, and shikonin by itself did not affect cell mitosis or apoptosis.

**Table 2:** Counts of mitotic and apoptotic cells in skin epidermal tissues.

	Average Mitotic Cells	Average Apoptotic Cells
DMSO	2.4±1.7^#^	1.8±0.8^#^
TPA	4.4±2.0*	2.8±0.9*
SKN	2.6±1.3^#^	2.2±0.8
SKN+TPA	2.1±0.9^#^	1.7±1.2^#^

Number of mitotic/apoptotic cells per 100 cells. DMSO, DMSO-treated group; TPA, DMBA/TPA-treated group; SKN, shikonin-treated group; SKN+TPA, shikonin plus DMBA/TPA-treated group.

^#^p<0.05 compared with the TPA group;

*p<0.05 compared with the DMSO group.

### PKM2 activities were not suppressed by shikonin in carcinogen-treated animal tissues

Since shikonin has been identified as a PKM2 inhibitor [13] and it inhibits PKM2 activation in TPA-treated skin epidermal JB6 cell [14], PKM2 activities were measured using pooled Whole Cell Lysate samples in each treatment group. As shown in S1 Fig, carcinogen activated PKM2; although PKM2 activity was suppressed by shikonin alone, it was not suppressed in the carcinogen-treatment group.

### Antibody microarray analysis

To identify potential shikonin targets, antibody microarray analysis was performed using pooled Whole Cell Lysate samples isolated from skin epidermal tissues at the end of the skin carcinogenesis study. The original images of the array slides were shown in S2 Fig. Quantified results were normalized to the internal control (actin) and these ratios were used for calculation. Positive hits were determined when the protein expression levels were increased more than 2-fold by DMBA/TPA treatment whereas these increases were significantly suppressed by shikonin.

**Fig 2:**
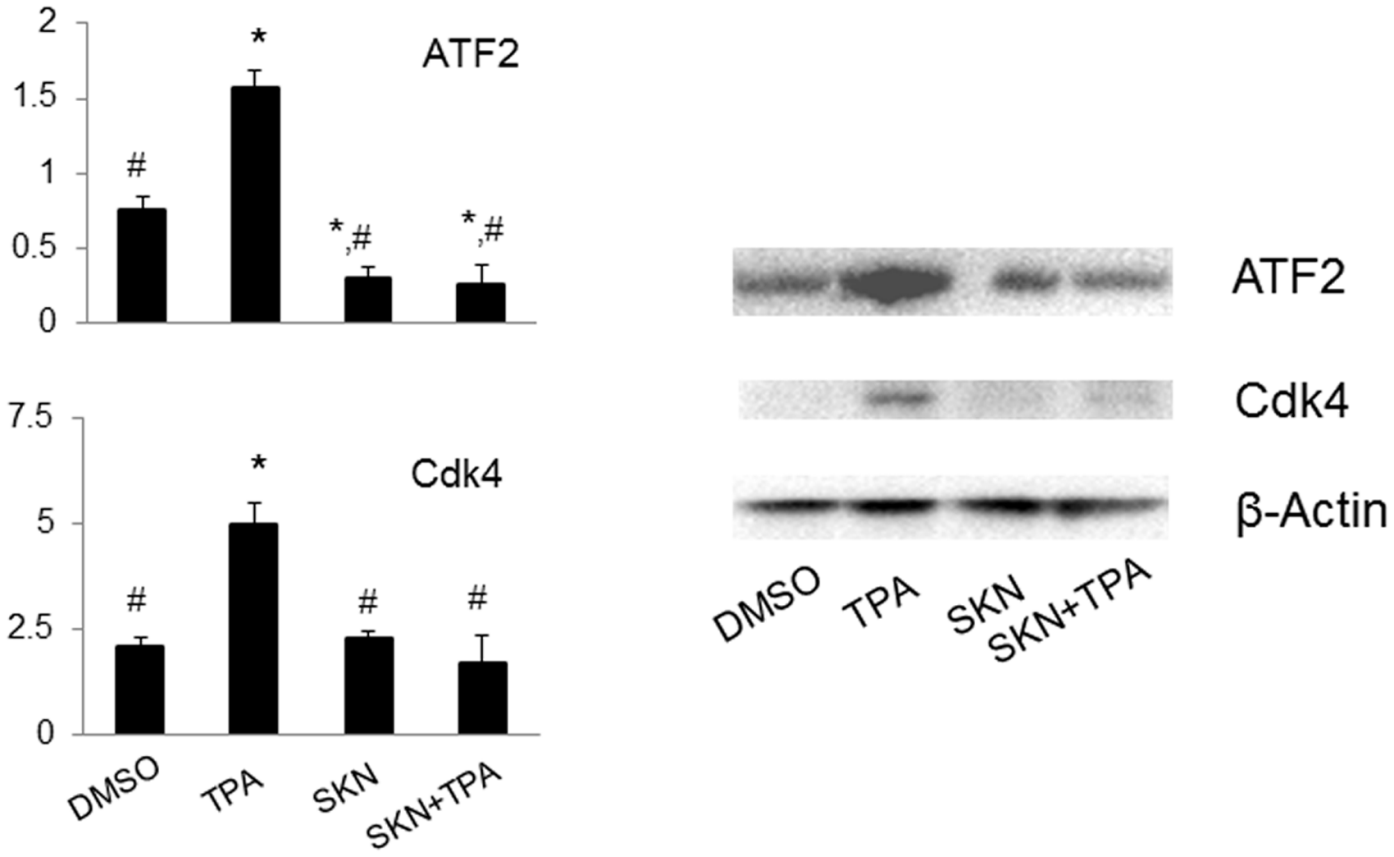
Detection of the expression levels of ATF2 and Cdk4 in mouse skin epidermal tissues at the end of the skin carcinogenesis study. Results were obtained from both the antibody microarray analysis (left panel) and Western blot analysis (right panel). Tissues from each individual mouse were pooled together and there were four repeats in each data group. DMSO, DMSO-treated group; TPA, DMBA/TPA-treated group; SKN, shikonin-treated group; SKN+TPA, shikonin plus DMBA/TPA-treated group. *, p<0.05 compared with the DMSO Group; #, p<0.05 compared with the TPA group.

In details, these targets include two kinases involved in cell proliferation and migration: JNK and Actopaxin (S3 Fig); four proteins involved in apoptosis: Bcl-10, caspase 3, 6, and 7 (S4 Fig); the transcription factor ATF2, and one of its target gene, the cell cycle regulator Cdk4 (Fig 2, left panel). The microarray results on ATF2 and Cdk4 were further confirmed using Western blot analysis (Fig 2, right panel). These two shikonin targets become the focus of the following studies.

### Shikonin decreased the nuclear levels of ATF2 and knockdown of ATF2 suppressed the expression levels of Cdk4 and Fra-1

**Fig 3:**
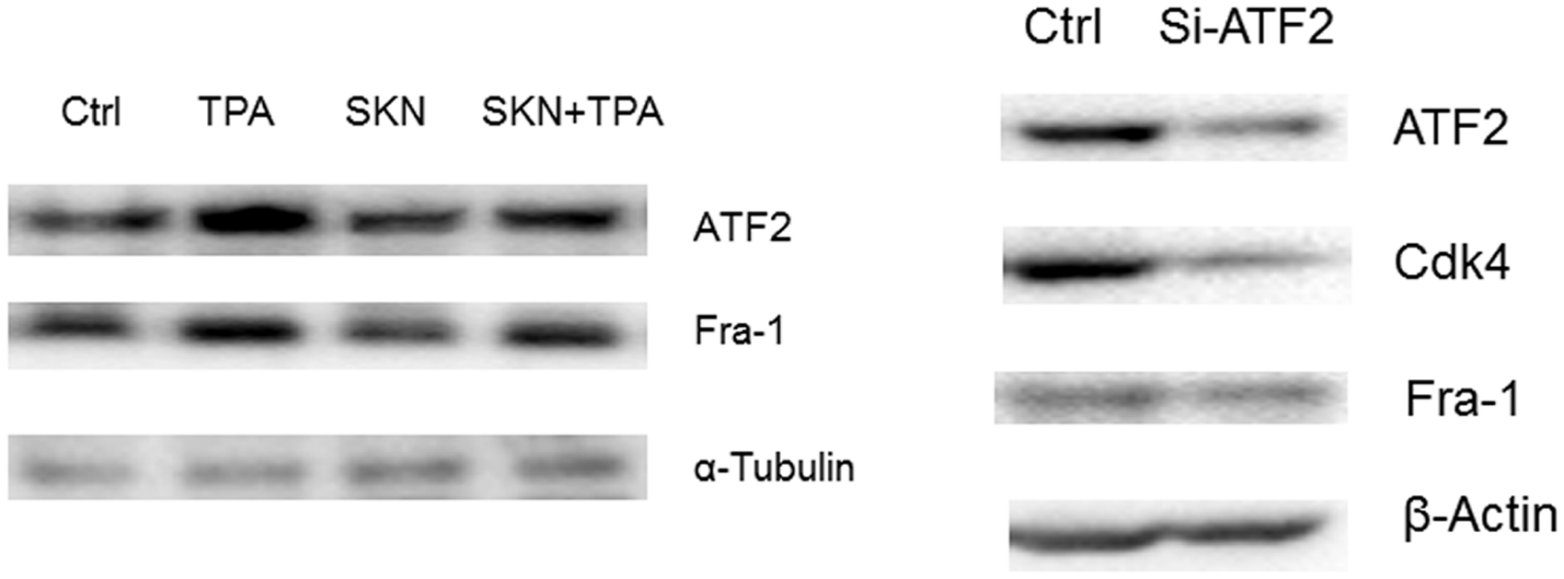
Detection of the expression levels of ATF2 and Cdk4 in mouse epidermal JB6 P+ cells. Left panel, JB6 cells were pretreated with shikonin (1 μM) for 30 min following by TPA (5 nM) treatment for 24 h. Nuclear extract was prepared and used for Western blot analysis. Right panel, JB6 P+ cells were transfected with siRNAs for 48 h and whole cell lysate was prepared for Western blot analysis.

Since ATF2 is a transcription factor, the nuclear levels of ATF2 were detected after shikonin treatment. The promotable skin epidermal JB6 P+ cells were treated with shikonin or the tumor promoter TPA. As shown in Fig 3 (left panel), TPA induced increases in ATF2 as well as Fra-1, a key subunit of activator protein 1 (AP-1) in the nucleus. To further verify the role of AFT2 in regulating Cdk4 and Fra-1, knockdown of ATF2 was performed using a siRNA approach in JB6 P+ cells. As shown in Fig 3 (right panel), the expression levels of Cdk4 and Fra-1 were both suppressed in ATF2 knockdown cells.

## Discussion

Shikonin, the naphthoquinone pigment isolated from *Lithospermum erythrorhizon* is the active component of a traditional Chinese medicine, which has been used to treat inflammation-related diseases and HIV-1 infection [1]. Its anti-tumor activity is reported largely due to induction of apoptosis in human cancer cells, including HL60 human premyelocytic leukemia cell line [2], hepatoma cells [4], colon cancer cells [5], melanoma cells [6], breast cancer cells [7], non-small cell lung cancer cells [8] and bladder cancer cells [9]. Shikonin is also reported to inhibit the growth of prostate cancer PC-3 cells [3]. Induction of apoptosis through coordinative modulation of the Bcl-2 family, p27, and p53, release of cytochrome *c*, and sequential activation of caspases in human colorectal carcinoma cells [5] was also reported. Similarly, shikonin can sensitize drug resistant cancer cells to treatment since it targets drug resistant genes [21]. Unlike the above studies, shikonin does not cause apoptosis in mouse skin epidermal tissues in the multistage skin carcinogenesis mouse model. This might be due to that the concentration of shikonin used in this study and/or shikonin is applied to a chronic tumor model.

Anti-inflammation is another possible mechanism of its anti-tumor effect. In transformed human mammary epithelial cells, shikonin has been shown to inhibit TPA-induced cyclooxygenase-2 (COX-2) activation, which is mediated by suppression of MAPK signaling [22].

Shikonin first showed chemopreventive activity in azoxymethane-induced intestinal carcinogenesis in rats via a dietary approach [15]; however, further studies are needed to test chemoprevention in other cancer models and to reveal the molecular mechanism.

Our previous study using a tumor promotion model [14] has shown that shikonin can suppress cell transformation which is associated with reduced glycolysis. This finding suggests that shikonin’s anti-tumor promotion works through inhibition of PKM2 activity. In the current study, although PKM2 activity is inhibited by shikonin alone, it is still increased when carcinogens are present. The PKM2 activity was measured at the end of the skin carcinogenesis study and the shikonin+TPA group also developed tumors. We cannot rule out the possibility that shikonin might be able to inhibit PKM2 during the early stage of skin carcinogenesis. It will be interesting to monitor PKM2 levels via a reporter system throughout the whole stage of skin carcinogenesis.

As a natural product, shikonin may also have other targets and/or affect other signaling pathways. The targets identified using the antibody microarray analysis provides interesting candidates.

Activating transcription factor 2 (ATF2) is one of the transcription factors whose induction is inhibited by shikonin. ATF2 is a member of the ATF/cyclic AMP-responsive element binding protein family of transcription factors. It has been implicated in malignant and non-malignant skin tumor developments. Inhibiting ATF2 suppresses melanoma development [23]. ATF2 can be phosphyralated by JNK and contributes to AP-1 binding activity [24,25]. However, ATF2 also exerts tumor suppressive activity in chemically induced skin carcinogenesis model [26], and protein kinase-c ε (PKCε) shifts ATF2 towards to tumor promotion. An early study [27] has indicated that in our skin carcinogenesis model, it is PKCε, not other PKCs, that is activated during skin carcinogenesis, which suggest that ATF2 may function as an oncogene in this study, and shikoin may exert its tumor suppressive effect via PKCε and JNK and their downstream target ATF2.

Cdc activates cyclin-dependent kinases (Cdks) by the removal of phosphates from residues in the Cdk active site. Cdk4 is part of the cyclin-dependent kinase family, and overexpression of Cdk4 in mouse skin results in increased susceptibility to squamous cell carcinoma development in a chemically induced carcinogenesis model [28].

Actopaxin (α-parvin), a kinase regulating cell adhesion and motility, is also suppressed by shikonin during skin carcinogenesis. Actopaxin is a paxillin, integrin-linked kinase, and F-actin binding focal adhesion protein. Actopaxin has been found to contribute to the regulation of matrix degradation and cell invasion in osteosarcoma and breast cancer cells. Rac1 seems to be required for actopaxin-induced motility [29].

DNA damage, oncogenic stress, and oxidative stress induced by chemical carcinogens can also lead to growth arrest and apoptosis during skin carcinogenesis. Caspase recruitment proteins and caspases which are up-regulated in this model might be responsible for this apoptotic event. Bcl-10 (B-cell lymphoma/leukemia 10) contains a caspase recruitment domain. Increased expression of active caspase 3 and 6 has been reported in melanoma [30] and mouse skin carcinogenesis models [19,20]. The role of this apoptotic event can be a consequence of cancer development which may provide growth advantage for the growth of other cancer cells [31].

In summary, the results from this study suggest that shikonin is effective in inhibiting chemically-induced skin carcinogenesis which is mediated largely by inhibiting cell proliferation during skin carcinogenesis. The potential target identified in this study, ATF2, will be examined in future experiments.

## Supporting Information

S1 Fig Detection of PKM2 activity in mouse epidermal tissues at the end of the skin carcinogenesis study. Whole cell lysate from each mouse tissue was pooled together and there were four repeats in each data group. DMSO, DMSO-treated group; TPA, DMBA/TPA-treated group; SKN, shikonin-treated group; SKN+TPA, shikonin plus DMBA/TPA-treated group. *, p<0.05 compared with the DMSO Group.
(DOCX)

S2 Fig Antibody microarray slides scanned by a fluorescence scanner. Whole cell lysate was pooled together in each data group. DMSO, DMSO-treated group; TPA, DMBA/TPA-treated group; SKN, shikonin-treated group; SKN+TPA, shikonin plus DMBA/TPA-treated group.
(DOCX)

S3 Fig Detection of the expression levels of Actopaxin and JNK in mouse epidermal tissues at the end of the skin carcinogenesis study. Results were obtained from the antibody microarray analysis. Tissues from each individual mouse were pooled together and there were four repeats in each data group. DMSO, DMSO-treated group; TPA, DMBA/TPA-treated group; SKN, shikonin-treated group; SKN+TPA, shikonin plus DMBA/TPA-treated group. *, p<0.05 compared with the DMSO Group; #, p<0.05 compared with the TPA group.
(DOCX)

S4 Fig Detection of the expression levels of Bcl-10 and caspases in mouse epidermal tissues at the end of the skin carcinogenesis study. Results were obtained from the antibody microarray analysis. Tissues from each individual mouse were pooled together and there were four repeats in each data group. DMSO, DMSO-treated group; TPA, DMBA/TPA-treated group; SKN, shikonin-treated group; SKN+TPA, shikonin plus DMBA/TPA-treated group. *, p<0.05 compared with the DMSO Group; #, p<0.05 compared with the TPA group.
(DOCX)
