# Supplementary material for: Shikonin Suppresses Skin Carcinogenesis via Inhibiting Cell Proliferation
Source: PLoS One. 2015 May 11;10(5):e0126459. doi: 10.1371/journal.pone.0126459 (PMC4427333; doi:10.1371/journal.pone.0126459)
Supplement: S2 Fig — Whole cell lysate was pooled together in each data group. DMSO, DMSO-treated group; TPA, DMBA/TPA-treated group; SKN, shikonin-treated group; SKN+TPA, shikonin plus DMBA/TPA-treated group. (DOCX) [file pone.0126459.s002.docx]

**S2 Fig. Antibody microarray slides scanned by a fluorescence scanner.** Whole cell lysate was pooled together in each data group. DMSO, DMSO-treated group; TPA, DMBA/TPA-treated group; SKN, shikonin-treated group; SKN+TPA, shikonin plus DMBA/TPA-treated group.
