# Supplementary material for: Shikonin Suppresses Skin Carcinogenesis via Inhibiting Cell Proliferation
Source: PLoS One. 2015 May 11;10(5):e0126459. doi: 10.1371/journal.pone.0126459 (PMC4427333; doi:10.1371/journal.pone.0126459)
Supplement: S4 Fig — Results were obtained from the antibody microarray analysis. Tissues from each individual mouse were pooled together and there were four repeats in each data group. DMSO, DMSO-treated group; TPA, DMBA/TPA-treated group; SKN, shikonin-treated group; SKN+TPA, shikonin plus DMBA/TPA-treated group. *, p<0.05 compared with the DMSO Group; #, p<0.05 compared with the TPA group. (DOCX) [file pone.0126459.s004.docx]

**S4 Fig. Detection of the expression levels of Bcl-10 and caspases in mouse epidermal tissues at the end of the skin carcinogenesis study.** Results were obtained from the antibody microarray analysis. Tissues from each individual mouse were pooled together and there were four repeats in each data group. DMSO, DMSO-treated group; TPA, DMBA/TPA-treated group; SKN, shikonin-treated group; SKN+TPA, shikonin plus DMBA/TPA-treated group. *, p<0.05 compared with the DMSO Group; #, p<0.05 compared with the TPA group.
